# Cytosolic, Autocrine Alpha-1 Proteinase Inhibitor (A1PI) Inhibits Caspase-1 and Blocks IL-1β Dependent Cytokine Release in Monocytes

**DOI:** 10.1371/journal.pone.0051078

**Published:** 2012-11-30

**Authors:** Yonggang Wang, Yong He, Bindu Abraham, Farshid N. Rouhani, Mark L. Brantly, Dorothy E. Scott, Jennifer L. Reed

**Affiliations:** 1 Center for Biologics Evaluation and Research, Food and Drug Administration, Bethesda, Maryland, United States of America; 2 Department of Medicine, University of Florida, Gainesville, Florida, United States of America; INSERM, France

## Abstract

**Rationale:**

Activation state-dependent secretion of alpha-1 proteinase inhibitor (A1PI) by monocytes and macrophages was first reported in 1985. Since then, monocytes and tissue macrophages have emerged as key sentinels of infection and tissue damage via activation of self-assembling pattern recognition receptors (inflammasomes), which trigger inflammation and cell death in a caspase-1 dependent process. These studies examine the relationship between A1PI expression in primary monocytes and monocytic cell lines, and inflammatory cytokine expression in response to inflammasome directed stimuli.

**Methods:**

IL-1 β expression was examined in lung macrophages expressing wild type A1PI (A1PI-M) or disease-associated Z isoform A1PI (A1PI-Z). Inflammatory cytokine release was evaluated in THP-1 monocytic cells or THP-1 cells lacking the inflammasome adaptor ASC, transfected with expression vectors encoding A1PI-M or A1PI-Z. A1PI-M was localized within monocytes by immunoprecipitation in hypotonic cell fractions. Cell-free titration of A1PI-M was performed against recombinant active caspase-1 in vitro.

**Results:**

IL-1 β expression was elevated in lung macrophages expressing A1PI-Z. Overexpression of A1PI-M in THP-1 monocytes reduced secretion of IL-1β and TNF-α. In contrast, overexpression of A1PI-Z enhanced IL-1β and TNF- α secretion in an ASC dependent manner. A1PI-Z-enhanced cytokine release was inhibited by a small molecule caspase-1 inhibitor but not by high levels of exogenous wtA1PI. Cytosolic localization of A1PI-M in monocytes was not diminished with microtubule-inhibiting agents. A1PI-M co-localized with caspase-1 in gel-filtered cytoplasmic THP-1 preparations, and was co-immunoprecipitated with caspase 1 from nigericin-stimulated THP-1 cell lysate. Plasma-derived A1PI inhibited recombinant caspase-1 mediated conversion of a peptide substrate in a dose dependent manner.

**Conclusions:**

Our results suggest that monocyte/macrophage-expressed A1PI-M antagonizes IL-1β secretion possibly via caspase-1 inhibition, a function which disease-associated A1PI-Z may lack. Therapeutic approaches which limit inflammasome responses in patients with A1PI deficiency, in combination with A1PI augmentation, may provide additional respiratory tissue-sparing benefits.

## Introduction

Since the first report in 1969 that alpha-1 proteinase inhibitor (A1PI) inhibits elastase [1], it has been widely accepted that excessive elastolytic activity underlies rapidly-progressing emphysema in patients with low plasma A1PI levels. Intravenous A1PI supplementation therapy, approved in 1987, specifically aims to restore the protease:antiprotease balance in lung tissue of such patients, particularly during respiratory infection when neutrophil degranulation is most prominent. However, pulmonary disease progresses even with intravenous A1PI supplementation, prompting the examination of additional mechanisms of A1PI-mediated lung tissue homeostasis.

Expression of elastase-inhibiting A1PI in lung macrophages and monocytes was initially reported in 1985 [2], although the biological significance was not understood. More recent observations in primary monocytes cultured ex vivo linked expression of aggregation prone, disease associated isoforms of A1PI with increased inflammatory cytokine expression, induction of apoptosis and/or autophagy, and endoplasmic reticulum (ER) stress-induced changes termed the unfolded protein response (UPR) [3]–[4]. How these and other changes in monocyte function may contribute to lung tissue damage in patients with A1PI deficiency has been incompletely explored.

Monocytes and tissue macrophages are key sentinels of infection and injury, in large part via activation of self-assembling pattern recognition receptors (inflammasomes) which trigger inflammation and cell death in a caspase-1 and IL-1β dependent process [5]–[6]. IL-1β in particular has emerged as a key regulator of stress-induced inflammation. Very recent data show that autocrine IL-1β signaling through the stress activated protein kinase (SAPK)/ Jun N-terminal kinase (JNK) pathway is required for inflammatory cytokine release in response to pathogen and damage associated inputs [7]. Similarly, IL-1β signaling through the JNK pathway has long been invoked in ER stress responses such as cytokine release and apoptosis [8]–[10]. Prompted by the observation of an inverse relationship between A1PI and IL-1β secretion in monocyte cell lines, in the current study we present evidence for a novel, intracellular role for monocyte/macrophage expressed wild type A1PI (A1PI-M) in antagonizing IL-1β secretion via caspase-1 inhibition, an activity which a disease associated A1PI isoform (A1PI-Z) appears to lack. Enhanced inflammatory cytokine release from monocytes expressing A1PI-Z was attenuated in cells lacking the inflammasome adaptor ASC, highlighting the role of inflammasome signaling in stress responses of monocytes. We present evidence that accumulation of A1PI-M in the monocyte cytosol occurs through retrograde transport through the ER, rather than through endocytosis of secreted protein. Our data demonstrate that in monocytes stably expressing A1PI-Z, a small molecule caspase-1 inhibitor blocks IL-1β release while high levels of exogenous, plasma derived A1PI-M do not, suggesting that uptake of exogenous A1PI-M inefficiently delivers inflammasome-inhibiting activity to the monocyte cytosol. Our data suggest that in patients with lung disease associated with A1PI deficiency, loss of inflammasome-inhibiting activity of A1PI-M may heighten cytokine release and increase inflammatory responses to respiratory pathogen- and damage-associated signals. Inflammasome-inhibiting co-therapies, combined with A1PI augmentation, could potentially confer additional tissue-sparing benefits to these patients.

## Materials and Methods

### Human Cells and Tissues

All studies involving retrospective analyses of pre-existing, de-identified human tissues acquired at autopsy were reviewed and deemed HIPPA compliant by the Research Involving Human Subjects Committee, Food and Drug Administration, Department of Health and Human Services (approval 08–025B). Tissue banks in each institution used informed consent from family members for collection of tissue. Tissue banks further eliminated all patient identifiers and provided unique identification numbers for donated lung tissue prior to shipment. Lung tissue acquired at autopsy from adults without known lung disease were purchased from US Biomax. Postmortem lung tissues from infants with acute fatal RSV lower respiratory infection were provided by Dr. Luis Avendaño at Roberto del Rio Children's Hospital, Santiago, Chile. IRB approval was given by the Ethics Commission of the University of Chile School of Medicine. Signed consent for use of autopsy tissue was obtained from the parents of infants.

Lung tissues from age-matched infants who died of other causes were provided by the National Institute of Child Health and Development Tissue Bank in Baltimore, MD. The collection of lung tissue at the National Institute of Child Health and Development Tissue Bank was approved by the University of Maryland, Baltimore Institutional Review Board. The tissue bank is IRB approved to obtain either written or verbal consent from parents regarding the use of infant lung tissues collected at autopsy. Verbal consent was documented by either recording the telephone call to the parents, or by witness verification over the phone of the consent to donate.

Lung tissues from patients with advanced respiratory disease associated with A1PI deficiency and cystic fibrosis were provided by the National Disease Research Interchange and by Dr. Scott Randell at the Cystic Fibrosis Center, University of North Carolina School of Medicine, Chapel Hill, NC. The University of North Carolina Committee for the Rights of Human Subjects approved the collection and use of excess surgical pathology tissue, obtained at the time of lung transplant, from patients with advanced lung disease associated with cystic fibrosis. Tissue collection from adult patients with advanced lung disease and alpha-1 antitrypsin deficiency was approved by the University of Florida Health Center Institutional Review Board. Informed written consent was obtained from all the adult lung tissue donors.

Studies involving elutriated monocytes acquired from healthy adult volunteers were approved by the Research Involving Human Subjects Committee, Food and Drug Administration, Department of Health and Human Services (IRB approval 03–120B). Elutriated monocytes acquired from healthy adult volunteers providing informed consent were collected under a protocol approved by the Institutional Review Board of the Clinical Center, National Institutes of Health, Bethesda, MD (99-CC-0168). Primary peripheral blood monocytes and lung macrophages expressing wtA1PI or Z-A1PI were obtained from outpatient volunteers 18 years and older at the University of Florida Alpha-1 Antitrypsin Deficiency Clinic. Written informed consent was obtained from all donors. The protocol was approved by University of Florida Health Center Institutional Review Board (IRB 008–2007).

### Chemicals and Reagents

Phorbol myristate acetate (PMA), LPS, PGN, *S. typhimurium* flagellin, muramyl dipeptide (MDP), and nigericin were purchased from Invivogen (San Diego, CA). Full-length human A1PI clone (BC011991.1) was obtained from Open Biosystems (Lafayette, CO). Recombinant capase-1 and irreversible caspase-1 inhibitor Ac-YVAD-CHO were purchased from Enzo Life Science (Farmingdale, NY). Chicken anti-A1PI and A1PI Standard were from Genway (San Diego, CA). Rabbit anti-A1PI antibody was from DAKO (Carpinteria, CA). Rabbit anti-caspase-1 and rabbit anti-CD44 antibodies were purchased from Cell Signaling Technology (Danvers, MA). Mouse anti-β-actin antibodies were purchased from Thermo Scientific (Rockford, IL). ELISA reagents for cytokine detection were obtained through R and D Systems (Minneapolis, MN). Anti-CD68 antibody was purchased from Santa Cruz Biotechnology (Santa Cruz, CA). A commercially licensed preparation of purified, plasma-derived A1PI was used for in vitro studies. Bovine serum albumin (BSA) and bovine gelatin control proteins were purchased from Sigma (St. Louis, MO). LPS induced cell death was assessed using Cell Death Detection ELISA kit (Roche, Indianapolis, IN) according to manufacturer’s instructions.

### Immunohistochemistry

Formalin-fixed, paraffin-embedded lung tissues were sectioned and processed through xylene to remove paraffin. Heat-induced epitope retrieval was performed prior to immunohistochemistry (IHC). Primary antibodies reactive with A1PI or CD68 were used according to manufacturer recommendations. Primary antibodies were detected with biotinylated secondary antibodies (Jackson Immunoresearch, West Grove, PA) and streptavidin/horseradish peroxidase conjugate (GE Healthcare, Piscataway, NJ) with peroxidase substrate (Sigma, St. Louis, MO).

### Cell Culture

Primary elutriated monocytes and cultured cell lines THP-1, U937, 28SC (ATCC), THP-1 lacking ASC (Invivogen, San Diego, CA) and MonoMac-6 (MM6; German Collection of Microorganisms and Cell Cultures, Mascheroder, Braunschweig, Germany) were cultured and maintained in RPMI 1640 supplemented with 2 mM GlutaMAXTM, 10 mM HEPES, 1 mM sodium pyruvate, 4.5 g/l glucose, 100 µg/ml streptomycin, 100 U/ml penicillin, and 10% heat–treated fetal calf serum (FCS; reagents supplied by Invitrogen, Grand Island, NY). For differentiation of monocytes, PMA was used at 1.6 nM final concentration.

### Transfection Studies and Quantitative PCR (Q-PCR)

Full-length human A1PI-M was subcloned into *Nhe1-HindIII* sites of pCDNA3.1 vector with a FLAG tag on its carboxyl terminal with the following primers: ^5^′ CTAGCTAGCGCGATGCCGTCTTCTGTCTCGTGGGGC^3^′; ^5^′ CCCAAGCTTTCAATGGTGATGGTGATGATGCTTGTCATCGTCATCCTTGT AATCTTTTTGGGTGGGATTCACCAC^3^′; For A1PI-Z, the following primers were used:^5^′GTTTTTAGAGGCCATACCCAGGTCTATCCCCCCCGAGGTCAA^3^′;^5^′TTGACCTCGGGGGGGATAGACCTGGGTATGGCCTCTAAAAAC^3^′;

Nucleofection technology (Nucleofector II, Lonza, Basel, Switzerland) was used for monocyte cells transfection according to instructions provided by the manufacturer. In brief, 1×10^6^ cells were nucleofected with 0.5 µg plasmid DNA, and protocol V-001 was used for THP-1 transfection. After nucleofection cells were cultured 8 to 24 hours before further treatment. For stable expression of FLAG-A1PIs, cells were maintained in G418 (800 µg/ml; Invitrogen, Grand Island, NY) for selection. For Q-PCR, total RNA was prepared usint Trizol LS Reagent (Invitrogen, Grand Island, NY) and cDNA was prepared using an RT^2^ First Strand kit (Qiagen, Valencia, CA) according to manufacturer’s instructions. Amplification of IL-1.

cDNA was performed using the following primers: ^5^′ TTCTTTCCCTTCATCTTTGA^3^′; ^5^′ACCACTTGTTGCTCCATATC^3^′. For amplification of control β-actin cDNA, the following primers were used: ^5^′AGCATTGCTTTCGTGTAAAT^3^′; ^5^′AGACCAAAAGCCTTCATACA^3^′. Forty cycles of PCR were performed using iTaq Universal SYBR Green Supermix (Biorad, Hercules, CA) according to manufacturer’s instructions, and analyzed using the Mx3000P RT-PCR system and software (Agilent, Santa Clara, CA).

### Biochemical Methods

To prepare the cell lysate for gel filtration, PMA stimulated THP-1 or unstimulated U937 cells were harvested and washed twice in cold phosphate-buffered saline, followed by swelling in 4 volumes of ice-cold hypotonic buffer: 20 mM HEPES-KOH [pH 7.5], 10 mM KCl, 1.5 mM MgCl2, 1 mM Na EDTA, 1 mM Na EGTA, 0.1 mM AEBSF (all from Sigma, St. Louis, MO) and protease inhibitor cocktail (Roche, Indianapolis, IN). After incubation on ice for 30 min, cells were disrupted by passage 15 times through a 22 gauge needle (Fisher Scientific, Pittsburgh, PA). Cell lysates were centrifuged at speed of 12,000 RPM for 30 minutes, and the supernatants were further cleared via filtration (0.45 µM) before loading onto Sepharose 6 (GE Healthcare, Pitscataway, NJ) equilibrated with buffer (20 mM HEPES-KOH [pH 7.5], 150 mM NaCl, 5% glycerol). The samples were separated by NuPAGE 4–12% protein gels, and transferred onto nitrocellulose membranes (Invitrogen, Grand Island, NY) for immunoblot. Dilutions of primary and HRP-conjugated secondary antibodies were prepared in 5% BSA blocking buffer. Incubations were performed overnight at 4°C and at room temperature for 1 hour, respectively. Visualization of reactive protein bands was performed using enhanced chemiluminescence detection reagents (GE Healthcare, Piscataway, NJ), and quantification was performed using Photoshop CD5 software.

### Protein Identification by LC–MS/MS

The Coommassie stained protein bands corresponding to 56 and 45 kDa was excised from the SDS–PAGE gel, and in-gel digestion with trypsin and extraction of peptides from gel slices were performed as described previously [11]. The tryptic peptides were reconstituted and analyzed on a linear ion trap Fourier transform ion cyclotron mass spectrometer (Thermo Electron, San Jose, CA) by electrospray ionization as described previously [12]. MSMS data were acquired (Xcalibur 2.0) and processed (Bioworks) to obtain (dta) files. Database searching was performed using the MASCOT database search engine (http://www.matrixscience). The following parameters were used: database, SPTREMBLE; taxonomy, *human*); one missed cut cleavage; methinone oxidation as variable modification and charge states of +2, +3, and +4. A window of 10 ppm for mass accuracy for precursor ions and 0.6-Da mass accuracy for MS-MS data was chosen. The results were parsed on Scaffold (Proteome Software, Portland, OR). The proteins identified were chosen by atleast two peptides and >95% peptide probability (based on MASCOT and X! Tandem scores).

### A1PI Deglycosylation Assay

Clarified hypotonic cytosolic samples or plasma-derived A1PI were denatured and treated with PNGase F (New England Biolabs, Ipswich, MA) according to manufacturer’s instructions. The samples were analyzed by SDS-PAGE and immunoblot as described above.

### Effects of A1PI on Caspase-1 Activity in vitro

Recombinant purified caspase-1 was prepared in reaction buffer provided by the manufacturer: 50 mM HEPES, pH7.4, 100 mM NaCl, 0.1 % CHAPS, 10 mM DTT, 1 mM EDTA, 10 % glycerol. Graded amounts of purified plasma-derived A1PI or bovine serum albumin or a caspase-1 inhibitor were added, followed by a fluorescent caspase-1 specific substrate (Ac-YVAD-AMC, Enzo Life Science, Farmingdale, NY). Conversion of the substrate at 30°C was measured over a time course using a 96 well plate reader (Molecular Devices, Sunnyvale, CA). A1PI inhibition of caspase-1 activity was measured and calculated in duplicate for a three independent experiments.

### Interactions of Caspase-1 with A1PI in vitro

Plasma-derived A1PI-M or gelatin control protein were crosslinked to NHS-ester agarose beads (GE Healthcare, Piscataway, NJ). Recombinant purified caspase-1 was incubated with 20 μ l of beads in the manufacturer’s recommended reaction buffer. After 1 h at 4°C with rotation, the beads were pelleted, washed with reaction buffer containing 1% NP40 (Sigma, St. Louis, MO), and eluted with 25 μ l of 0.1 M glycine, pH 2.3 (Sigma, St. Louis, MO). Samples were analyzed by immunoblot as described above.

### Statistical Analysis

Statistical differences between groups were determined by t-test using GraphPad Prism software (La Jolla, CA). All of the data are expressed as mean ± SD, with significance defined as P ≤ 0.05.

## Results

**Figure 1:**
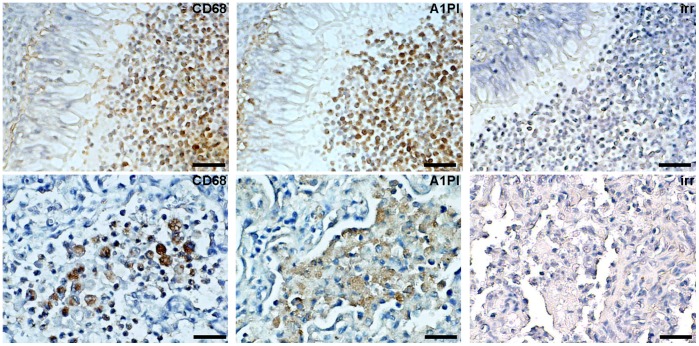
Cell Associated A1PI Detection in Human Lung Tissue. Lung tissue acquired at autopsy from patients with advanced lung disease associated with cystic fibrosis (n = 8, top row) and acute fatal respiratory syncytial virus infection (n = 9, bottom row). Representative detection of macrophage antigen CD68 (left panels), A1PI (middle panels), or irrelevant species-matched control antibody (right panels) are shown. Bar = 30 µm top row, 15 µm bottom row.

**Figure 2:**
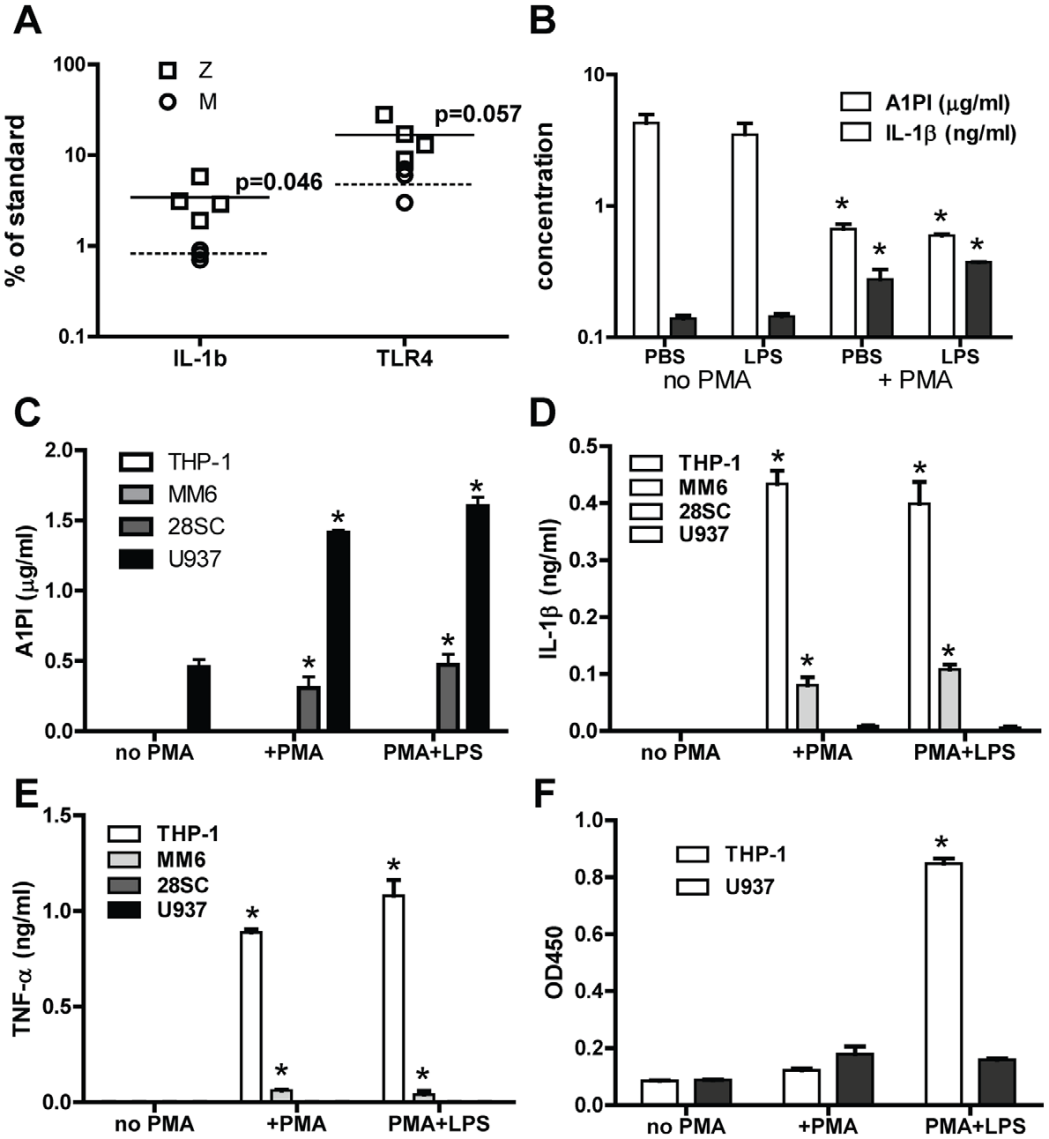
A1PI and Cytokine Secretion in Cultured Monocytes. A) IL-1β and TLR4 transcripts were evaluated by quantitative PCR in alveolar macrophages acquired by bronchoalveolar lavage from PIZZ (squares) or PIMM (circles) outpatient volunteers (n = 3–4). Bars indicate the mean values for PIZZ (filled bar) and PIMM (dashed bar) samples. B) Elutriated peripheral blood monocytes of healthy volunteers (n = 3) were cultured 5 days with or without PMA and LPS stimuli as indicated. Secreted A1PI and IL-1β were detected in collected supernatants by ELISA. C-E) Monocytic cell lines were cultured 5 days with or without PMA and LPS stimuli as indicated. Secreted A1PI (C), IL-1β (D), and TNF-α (E) were detected in collected supernatants by ELISA. E) THP-1 and U937 cultured 4 days with PBS control or with PMA were washed, and LPS (100 ng/ml) was added to selected wells. Released nucleosome:DNA complexes were assessed in supernatants collected at 24 hours. Asterisks denote P≤0.05 compared with unstimulated control cells. For (A) through (E), representative results of 3 experiments are shown.

Expression of A1PI-M observed in BAL-acquired macrophages was proposed to provide additional, local protection against elastase-induced injury [13]. To understand whether macrophage A1PI contributions to lung tissue homeostasis might differ among different disease states, we performed immunohistochemical assessment of A1PI in tissues acquired at autopsy from patients with severe cystic fibrosis (CF) associated respiratory disease, acute fatal lower respiratory infection (LRI), lung disease associated with A1PI deficiency and controls without known lung disease (Fig. 1 and not shown). Accumulation of A1PI positive cells in the airway lumen was striking in both RSV-associated LRI of infancy and in CF, two diseases in which dampened innate responses of tissue macrophages to pathogens have been invoked in respiratory tissue destruction [14]–[16]. In both LRI of infancy and advanced lung disease associated with CF, cell-associated A1PI and the macrophage marker CD68 appeared colocalized in sequential sections (Fig. 1), consistent with macrophage expression of A1PI. We considered whether A1PI-M expression in macrophages might limit responses to respiratory pathogens and damage. In possible support of this idea, we found significantly increased expression of innate immune mediators IL-1β and TLR4 by quantitative PCR in uncultured alveolar macrophages acquired during a period of non-exacerbation from a small number of outpatient PiZZ volunteers, compared with PiMM volunteers (Fig. 2a). We therefore decided to further investigate potential links between macrophage A1PI expression and innate immune responses.

Lung tissue macrophages are difficult to acquire in large numbers for in vitro studies. However, lung macrophages derive from blood monocytes, which are more readily available for in vitro evaluation and have well characterized responses to innate stimuli. Therefore we expanded our work to include primary A1PI-M monocytes under well characterized culture conditions. In previous reports, A1PI-M expression was high in primary monocytes and significantly elevated in monocytes maintained in GM-CSF, compared with monocytes cultured with differentiation agents such as phorbol ester or M-CSF plus cytokines [13], [17]–[18]. We confirmed secretion of A1PI-M by unstimulated peripheral blood monocytes of healthy volunteers, at high baseline levels which were in some but not all donors augmented by GM-CSF and LPS addition (Fig. 2b and not shown). PMA-differentiated monocytes secreted significantly less A1PI, and released more IL-1β and TNF-α at baseline and in response to endotoxin compared with non-PMA treated cells (Fig. 2b). When we extended these studies to other commonly used human monocytic cell lines (U937, MonoMac6, 28SC, THP-1), we found some monocytes secreted A1PI and others did not under identical baseline treatment conditions (Fig. 2c), highlighting the phenotypic heterogeneity of transformed human monocytes. Notably, A1PI-M secretion in the four cell lines was inversely related to IL-1β and TNF- α release (Fig. 2d-2e) and LPS-induced cell death (Fig. 2f), measured by DNA:histone fragments released into the supernatant at 24 hours post-stimulus.

Our follow-up analysis focused on U937 cells, which secreted the most A1PI-M, and THP-1 cells which secreted the least A1PI-M. Confirming and extending previous findings [19], we observed IL-1β protein in cell lysates but not supernatants of U937 cells stimulated with a wide variety of pathogen- and damage-associated stimuli associated with NALP3 inflammasome activation (Fig. S1) [5], suggesting a possible block in IL-1β maturation and/or release in U937 cells. Conversely, THP-1 cells exhibited a high level of IL-1β release with PMA which was not increased with various Toll ligand exposures and nigericin, suggesting enhanced IL-1β processing and/or release in these cells (Fig. S1). Based on these initial findings we considered the possibility that heightened A1PI-M expression in monocytes might be causally linked to limited IL-1β release, possibly through reduced inflammasome activity.

**Figure 3:**
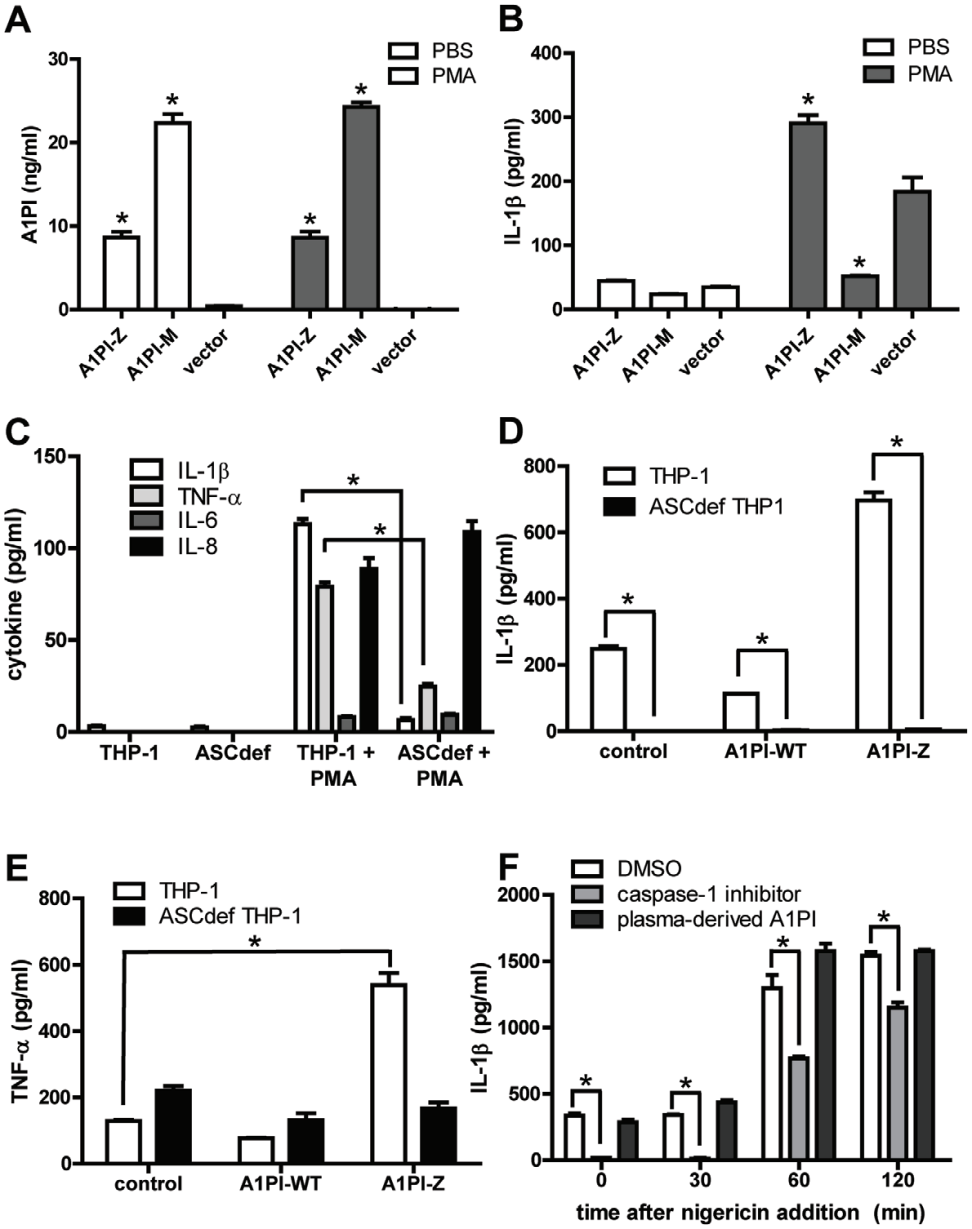
A1PI Impacts ASC Dependent IL-1β Release in Transfected Monocytes. A-B) THP-1 cells were nucleofected with plasmids encoding wild type A1PI (A1PI-M) or Z isoform A1PI (A1PI-Z). Cells were stimulated with PMA or PBS control for 5 days, and A1PI (A) and IL-1β (B) were detected in supernatant by ELISA. C) Secreted IL-6, IL-8, TNF-α and IL-1 β were detected in supernatants of THP-1 cells or THP-1 cells lacking ASC, incubated with PMA or PBS control for 5 days. D-E) THP-1 cells or THP-1 cells lacking ASC were nucleofected with vectors expressing A1PI-M or A1PI-Z, then challenged with PMA. Secreted IL-1β (D) and TNF- α (E) were detected in supernatants collected on day 5. F) THP-1 cells stably expressing A1PI-Z were pre-treated with caspase-1 inhibitor (40 µM) or plasma-derived A1PI (1 mg/ml), then PMA stimulated for 18 hours followed by 20 µM nigericin challenge. Secreted IL-1β was measured in supernatants collected up to 120 minutes post nigericin. Asterisks denote P≤0.05.

Aggregation, reduced secretion, lack of elastase inhibitory activity [20] and ER stress-mediated cytokine release [3] are reported features of disease-associated A1PI isoforms. We hypothesized that the ability to modulate release of IL-1β in monocytes might also differ between A1PI-M and disease-associated A1PI isoforms. To test this possibility, we studied THP-1 monocytes, the most commonly used cell model for the study of inflammasome activation and IL-1β release. THP-1 monocytes were separately nucleofected with an expression plasmid encoding either A1PI-Z or A1PI-M. Both A1PI isoforms were expressed and secreted at high levels in THP-1 cells at baseline, as demonstrated by detection in culture supernatant (Fig. 3a), although secretion of A1PI-Z was significantly less than A1PI-M. THP-1 cells nucleofected with the A1PI-M expression construct demonstrated reduced IL-1β release in response to 18 hour PMA treatment (Fig. 3b), compared with THP-1 cells nucleofected with a control plasmid. The reduction in released IL-1β appeared to be associated with downstream processing of the IL-1β protein, as similar levels of pro-IL-1β protein and IL-1β mRNA were found in A1PI-M overexpressing THP-1 cells and empty vector control cells (Suppl. Fig. 2). However, pro-IL-1β protein and mature IL-1β release were markedly enhanced in THP-1 cells overexpressing A1PI-Z (Fig. 3b and Fig. S2). THP-1 cells lacking the key inflammasome adaptor protein ASC demonstrated no IL-1β secretion and reduced TNF-α release with PMA stimulation, while IL-6 and IL-8 secretion remained unchanged (Fig. 3c). Transient expression of A1PI-Z augmented PMA-induced IL-1β and TNF- α release over controls in THP-1 cells, but not in THP-1 cells lacking ASC (Fig. 3d-e). We then tested whether exogenously delivered plasma-derived A1PI-M could regulate inflammasome activity in macrophages expressing mutant A1PI-Z. THP-1 cells expressing A1PI-Z were treated with a suboptimal dose of PMA, then challenged with a nigericin second signal to elicit maximal IL-1β secretion over a 2 hour time course according to published methods [21]. In control wells, IL-1β release increased approximately 5-fold; this increase was effectively reduced, particularly at early time points, by a cell permeable, irreversible caspase-1 inhibitor YVAD-CHO (Fig. 3f). In contrast, pretreatment of the cells with excess plasma-derived A1PI-M had no effect on IL-1β release (Fig. 3f).

**Figure 4:**
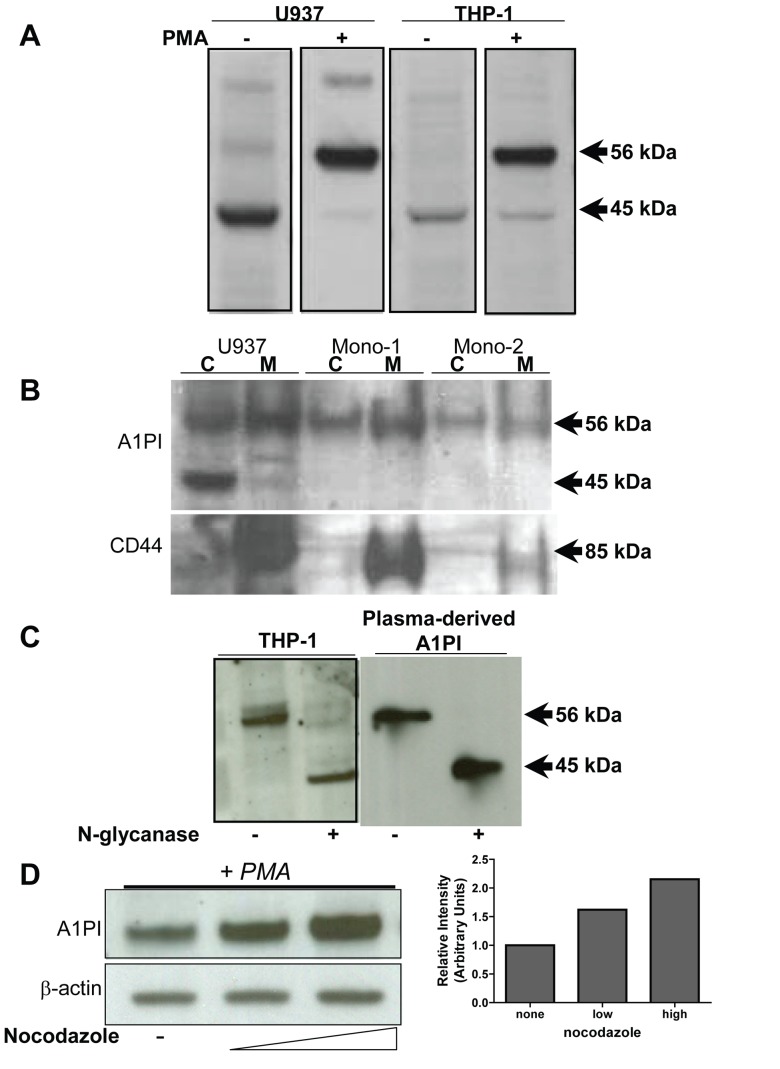
Cytosolic Monocyte-Expressed A1PI is Glycosylated. A) Immunoblot detection of A1PI in whole cell extracts of U937 and THP-1 monocytes with and without PMA stimulation. B) U937 cells or primary elutriated monocytes of two healthy donors (Mono-1, Mono-2) were lysed with hypotonic buffer. Cytosol (C) and membrane (M) fractions were analyzed by immunoblot for A1PI and the membrane associated protein CD44. C) THP-1 cytosolic samples prepared by hypotonic lysis and plasma-derived A1PI were incubated at 37°C with or without N-glycanase prior to immunoblot detection of A1PI. D) THP-1 cells were cultured with PMA with or without nocodazole stimulation for 18 hours. Cytosolic samples prepared by hypotonic lysis, and A1PI (upper panel) and β-actin loading control (lower panel) were analyzed by immunoblot. A1PI bands were quantified by densitometry (right).

Most previous studies of A1PI-M have focused on its extracellular anti-inflammatory actions. However, an A1PI-M mediated impact on inflammasome function might imply an intracellular localization. In support of a cytosolic localization of at least a portion of monocyte expressed A1PI-M, immunoblot analysis and mass spectroscopy demonstrated strong detection of A1PI-M in all four monocytic cell lines we studied, even under conditions where no secreted A1PI-M was detected in supernatants (Fig. 4a and data not shown). SDS-PAGE analysis of whole cell extracts demonstrated two protein bands at 56 and 45 kDa reactive with A1PI-directed polyclonal antibodies (Fig. 4a). We used hypotonic cell lysis to separate cytoplasmic and membrane fractions of U937 and THP-1 cell lines, and peripheral blood monocytes. Immunoblot analysis demonstrated A1PI-M in both the cytosolic and membrane fractions, while control protein CD44 was only observed with the membrane fractions (Fig. 4b). As observed with commercially available plasma-derived A1PI-M, macrophage-expressed A1PI-M was resolved to a single 45 kDa band with PNGase-F mediated removal of N-linked glycans (Fig. 4c), indicating that macrophage-expressed A1PI-M was processed at least through the Golgi and had been glycosylated. Based on the glycosylation result, we anticipated that A1PI-M in the cytosol had been accumulated through endocytic or pinocytic uptake of secreted protein. To test this hypothesis, we treated THP-1 cells with the microtubule inhibitor nocodazole, which broadly inhibits endocytosis and pinocytosis [22]–[23]. Unexpectedly, nocodazole failed to limit A1PI-M accumulation in THP-1 cytosol. Instead, nocodazole treatment increased A1PI-M detection in THP-1 cytosol, in either the presence or absence of PMA stimulus (Fig. 4d). Similar results were observed in primary peripheral blood monocytes of healthy volunteers (Fig. S3). In each case nocodazole treatment failed to attenuate, and sometimes increased, the detection of A1PI-M in the cytosolic fraction.

**Figure 5:**
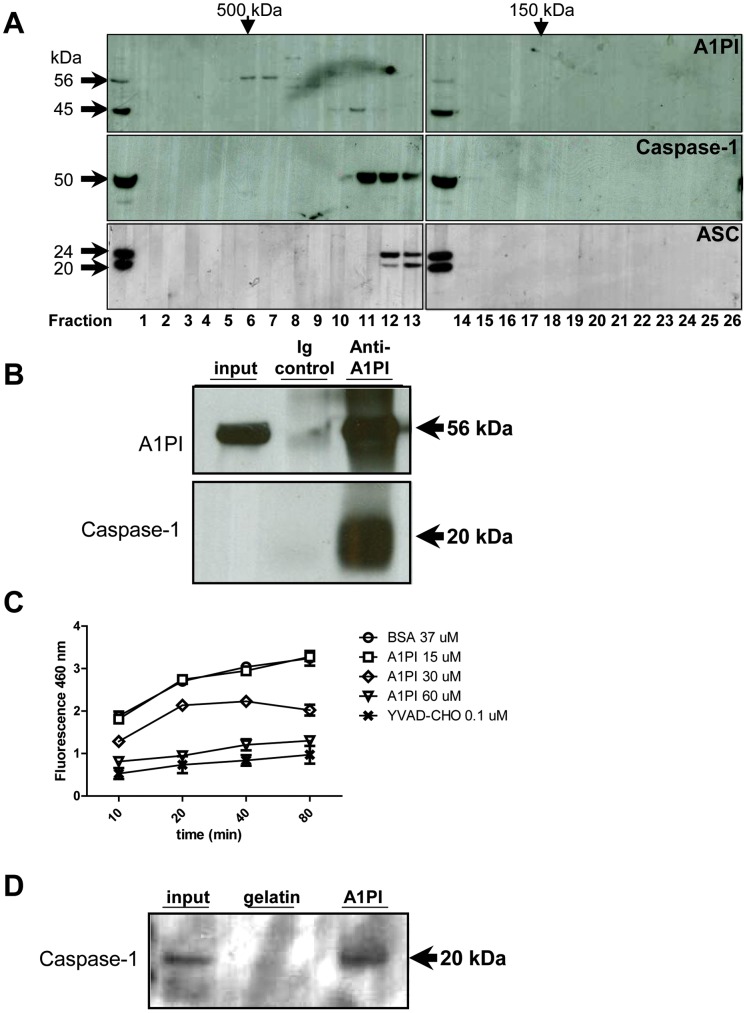
A1PI Interacts with Caspase-1 in Monocyte Cytosol. A) Cytosolic preparations of THP-1 cells activated 18 hours with PMA were applied to a Superdex S-200 column. Fractions 6 through 24 were analyzed by SDS-PAGE and immunoblot with antibodies directed to A1PI (top panel), caspase-1 (middle panel), and ASC (bottom panel). B) THP-1 cells activated overnight with PMA were pulsed for 1 hour with 20 µM nigericin before hypotonic lysis. Immunoprecipitation was performed using an irrelevant control IgG or A1PI-directed antibodies. Precipitated proteins were analyzed by immunoblot with anti-A1PI (top panel) or anti-caspase-1 (lower panel). C) Graded amounts of plasma derived A1PI, BSA control, or small molecule caspase-1 inhibitor, were coincubated with recombinant active caspase-1 prior to addition of a fluorescent peptide substrate. Reactions were incubated at 37 °C, and fluorescence over time in duplicate wells (mean and STD) were measured by platereader. D) Gelatin control protein or plasma derived A1PI were conjugated to agarose beads, then incubated with recombinant caspase-1. After extensive washing, proteins were eluted with SDS-PAGE buffer and analyzed by immunoblot for bands reactive with a caspase-1 directed polyclonal antibody.

We next evaluated whether cytosolic A1PI-M could interact with inflammasome components, using size fractionation analysis of hypotonic THP-1 preparations as previously described [24]. In THP-1 cells activated with PMA, A1PI-M was detected by immunoblot in cytosolic fractions containing molecular mass complexes from 200 to 500 kDa (Fig. 5a). We observed co-detection of A1PI-M and caspase-1 (Fig. 5a) in a subset of fractions, possibly consistent with a direct interaction of the two proteins. Co-immunoprecipitation analysis supported an interaction between A1PI-M and caspase-1. In hypotonic lysates of THP-1 cells pulsed with PMA plus nigericin, A1PI-directed antibodies co-immunoprecipitated the 56 kDa A1PI-M band plus a 20-kDa doublet reactive with caspase-1 directed polyclonal antibodies in immunoblot (Figure 5b). Potential interactions between A1PI-M and caspase-1 were further explored in a cell-free setting, using commercial preparations of purified plasma-derived A1PI-M and recombinant active caspase-1. In initial studies, recombinant caspase-1 formed a recoverable complex with plasma-derived A1PI-M, but not bovine gelatin, directly conjugated to beads (Fig. 5c). Similarly, A1PI-M, but not bovine albumin or gelatin, inhibited caspase-1 mediated in vitro conversion of a fluorescent substrate in a dose-dependent manner (Fig. 5d), providing additional evidence for cytoplasmic A1PI-M as a negative regulator of caspase-1 activity.

## Discussion

The current studies provide substantial evidence for a novel, intracellular role for A1PI-M in limiting caspase-1 activity and IL-1β release in monocytes. These results are similar to previous studies which invoke A1PI-M interactions with cytosolic executioner caspases 3 and 6 [25]–[26], and recently caspase-1 [27], as critical for staving off cell death in endothelial cells, epithelial cells, and cardiomyocytes. As in previous studies, we observed that an excess of A1PI was required to limit caspase activity in a cell free assay, which is highly reliant on the purity and potency of the enzyme preparation. One previous report showed very limited A1PI inhibition of caspase-1 enzymatic activity in a cell free enzyme assay, compared with caspase-3 and caspase-6 [26]. We note that the preparations of recombinant caspase-1 enzyme and reaction conditions used in our study were quite different and may underlie a sensitivity difference between the two assay systems. In addition the current studies were facilitated by the use of monocytes in which inflammasome signals are amplified, allowing easier observation of A1PI:caspase-1 interactions.

Importantly, unlike previous studies, our monocyte culture systems did not employ large amounts of exogenous plasma-derived A1PI-M. Instead, endogenous A1PI-M expression reduced IL-1β release in monocytes, indicating a new autocrine role for A1PI-M in limiting inflammatory responses. These results may add context to other recent studies of stress responses in primary monocytes [3], [7]. In a recent study, primary peripheral blood monocytes from patients expressing A1PI-Z demonstrated an increase in NFκB signaling under baseline conditions and increased LPS-induced IL-6, IL-8 and IL-10, compared with monocytes from healthy volunteers expressing A1PI-M [3]. A second recent study of peripheral blood monocytes from healthy volunteers demonstrated early, autocrine IL-1β release is required for optimal TNF- α, IL-6, IL-8 and IL-10 release after stimulus with MDP, a ligand for the intracellular innate immune receptor Nod2 [7]. Our current studies, which combined ER stress associated with transient A1PI-Z expression with a broad-spectrum activating agent (PMA) in a monocytic cell line, demonstrated that A1PI-Z expression increased only IL-1β and TNF- α release. Together these data suggest that macrophage expressed A1PI can modulate cytokines differently in different circumstances, based on the cell type, the type of stimulus encountered, and the dependence on autocrine IL-1β. Alternatively the artificial conditions we used may have masked a role for A1PI-M in modulating IL-6 and IL-8 release. Additional roles for autocrine A1PI-M in dampening innate immune responses may be revealed by expanding this study to primary monocytes and macrophages, and evaluating additional damage- and pathogen-associated stimuli.

Cytosolic compartmentalization of A1PI-M in monocytes appears to be a critical part of effective inflammasome inhibition. Pinocytosis or receptor mediated endocytosis were the probable mechanisms of A1PI-M delivery to the cytosol in previous studies of endothelial and epithelial cells. However our data suggest this is not the case for monocytes. Cytosolic A1PI-M was observed in cells which do not secrete A1PI-M, making uptake from the supernatant unlikely. In addition, broad-spectrum inhibition of endocytosis and pinocytosis with nocodazole failed to block cytosolic accumulation of A1PI-M, suggesting that delivery to the cytosol may have occurred via retrograde transport from the Golgi through the ER. In fact, cytosolic A1PI-M in monocytes modestly increased in some cases with nocodazole treatment, consistent with retrograde cycling of A1PI from the Golgi, a pathway which is enhanced by this drug [28]. To our knowledge, this would be the first example of a homeostatic function for a wild type, presumably properly folded protein delivered to cytosol through the retrograde transport pathway generally associated with removal of unfolded proteins. UPR and ERAD are adaptive mechanisms intended to preserve cell function and survival in the face of myriad environmental stressors [29]. Retrograde transport from the ER under stress conditions in theory provides a rapid means of shuttling cytoprotective intact proteins directly to the cytosol in a tightly regulated manner. This is clearly not the case for bulk solute processed through pinocytosis, or for proteins taken up through receptor-mediated endocytosis which are typically degraded in the lysosome. Although plasma-derived A1PI-M did interact with caspase-1 in cell free conditions, extracellular concentrations of plasma-derived A1PI-M up to 1 mg/ml did not provide additional caspase-1 blocking activity to cultured monocytes under the experimental conditions we used. Studies are in progress to investigate whether even higher concentrations of extracellular A1PI and/or alternative activation conditions may enhance delivery of caspase-1 blocking activity into monocyte cytosol.

Our data could support a direct interaction between A1PI-M and caspase-1 in the cytosol, resulting in inhibition pro-IL-1β maturation. Elevated IL-1β secretion in A1PI-Z expressing monocytes could be consistent with a failure of A1PI-Z to limit caspase-1 activity, although the pro-inflammatory mechanism(s) of A1PI-Z in this system have not been completely evaluated. Whether misfolding of A1PI-Z might increase its rate of proteolysis in the cytosol, or could preclude binding to caspase-1, are hypotheses we are exploring in our current studies. The current data do not rule out pathogenic, gain-of-function interactions with innate immune receptors and/or the inflammasome which might augment IL-1β release. Additional activities of A1PI-M, for example as a scavenger of inflammasome components for removal via autophagosome [30], or as an intracellular modulator of reactive oxygen species [31], could also restrict IL-1β secretion in the context of whole cells [32].

Whatever the precise mechanism(s), inflammasome regulating activities of A1PI-M and A1PI-Z could have important implications for monocyte / macrophage function in health and disease. Autocrine IL-1β signaling in monocytes amplifies TLR-derived signals in vitro [7] and in vivo [33]–[34], through augmented stress-activated protein kinase (SAPK) / JNK signaling. Endogenous A1PI-M may serve to scale back and/or resolve inflammasome-dependent responses of monocytes and macrophages during respiratory infection [35]–[36], a function which may be diminished in patients with lung disease associated with A1PI deficiency. In addition, lipotoxicity-associated activation of the inflammasome augments ER stress in monocytes, leading to monocyte dysfunction in atherosclerosis and diabetes [37]–[41]. Impaired control of inflammasome activation in monocytes of patients with A1PI deficiency could contribute to diabetes susceptibility and atherosclerotic disease progression in these patients [42]–[43]. Inflammasome-dependent contributions of monocytes and macrophages to human COPD have not been fully elucidated, but are strongly suggested by animal models of smoke-induced and elastase-induced lung disease which display both IL-1R/MyD88 dependence and strong macrophage contributions [44]–[46]. Notably, lentiviral-based expression of A1PI-M in alveolar macrophages afforded marked protection against pulmonary damage in elastase- and cigarette smoke-induced emphysema models [47]. Respiratory tissue sparing was assumed to arise from anti-inflammatory and anti-apoptotic actions of secreted A1PI-M. Whether dampened macrophage responses to smoke and elastase challenge contributed to protection in these models was not explored. The possibility of long-term modulation of inflammasome-mediated responses in macrophages by targeted intracellular expression of A1PI can be addressed with additional studies.

An inflammasome activation signature has been noted in other chronic diseases featuring misfolded protein accumulation, including Alzheimer’s disease, amyotrophic lateral sclerosis and type II diabetes. In these disease settings, unfolded protein aggregates are proposed to gain novel activities such as lysosome damage and activation of pattern recognition receptors, resulting in pathogenic inflammasome activation [48]. How this handful of misfolded protein products should gain such deleterious functions, when many other misfolded proteins do not, remains a mystery. Chronic inflammasome activation and stress responses associated with misfolded SOD1, IAPP, and Aβ may alternatively be clues to important cytosolic roles for their wild type counterparts in scaling back inflammasome activity, facilitating inflammation resolution and preserving tissue homeostasis. Evaluation of the inflammasome-restricting activity of wild type versions of these proteins may offer valuable new therapeutic insights.

In closing, our studies provide evidence that loss of function of A1PI-M may underlie increased IL-1β secretion in monocytes, suggesting the possibility that loss of this activity contributes to inflammatory destruction of tissue in patients with A1PI deficiency. Therapeutic approaches that limit inflammasome responses, particularly during infection-associated exacerbations, could enhance the tissue-protective effects of A1PI augmentation in patients with lung disease associated with A1PI deficiency.

## Supporting Information

Figure S1 **IL-1**β **Release Compared in U937 and THP-1 Monocytes.** A) U937 cells were cultured for 5 days with PMA or PBS control, followed by addition of graded concentrations of muramyl dipeptide (MDP). After 18 hours, supernatants were collected, and cells were washed twice in PBS then lysed. IL-1β was assessed separately in supernatants (open bars) and cell lysates (filled bars) by ELISA. Asterisks denote increase from untreated cells, P≤0.05. B) THP-1 (connected circles) and U937 (connected squares) cultured with PMA for 5 days were pulsed for 18 hours with graded concentrations of TLR ligands peptidoglycan, flagellin, LPS, or MDP. IL-1β was assessed in supernatants by ELISA.
(TIF)

Figure S2 **Pro-IL-1β and IL-1β mRNA Detection in Transfected THP-1 Cells.** THP-1 cells stably transfected with plasmids encoding wild type A1PI (A1PI-M) or Z isoform A1PI (A1PI-Z), or empty vector control plasmid, were treated for 18 hours with PMA or DMSO vehicle. A) Level of IL-1β mRNA normalized to β-actin was detected by Q-PCR; mean of triplicate wells is shown. B) Level of pro-IL-1β protein in whole cell lysate was detected by ELISA; mean of duplicate wells is shown. Asterisks denote P≤0.05.
(TIF)

Figure S3 **Cytosolic A1PI is Detected after Endocytosis Inhibition in Primary Peripheral Blood Monocytes.** Primary elutriated monocytes from healthy adult volunteers were cultured with low or high concentrations of nocodazole plus PMA stimulation for 18 hours. A1PI was detected by immunoblot in cytosolic samples prepared by hypotonic lysis.
(TIF)
