# Supplementary figures and images for: Cytosolic, Autocrine Alpha-1 Proteinase Inhibitor (A1PI) Inhibits Caspase-1 and Blocks IL-1β Dependent Cytokine Release in Monocytes
Source: PLoS One. 2012 Nov 30;7(11):e51078. doi: 10.1371/journal.pone.0051078 (PMC3511367; doi:10.1371/journal.pone.0051078)

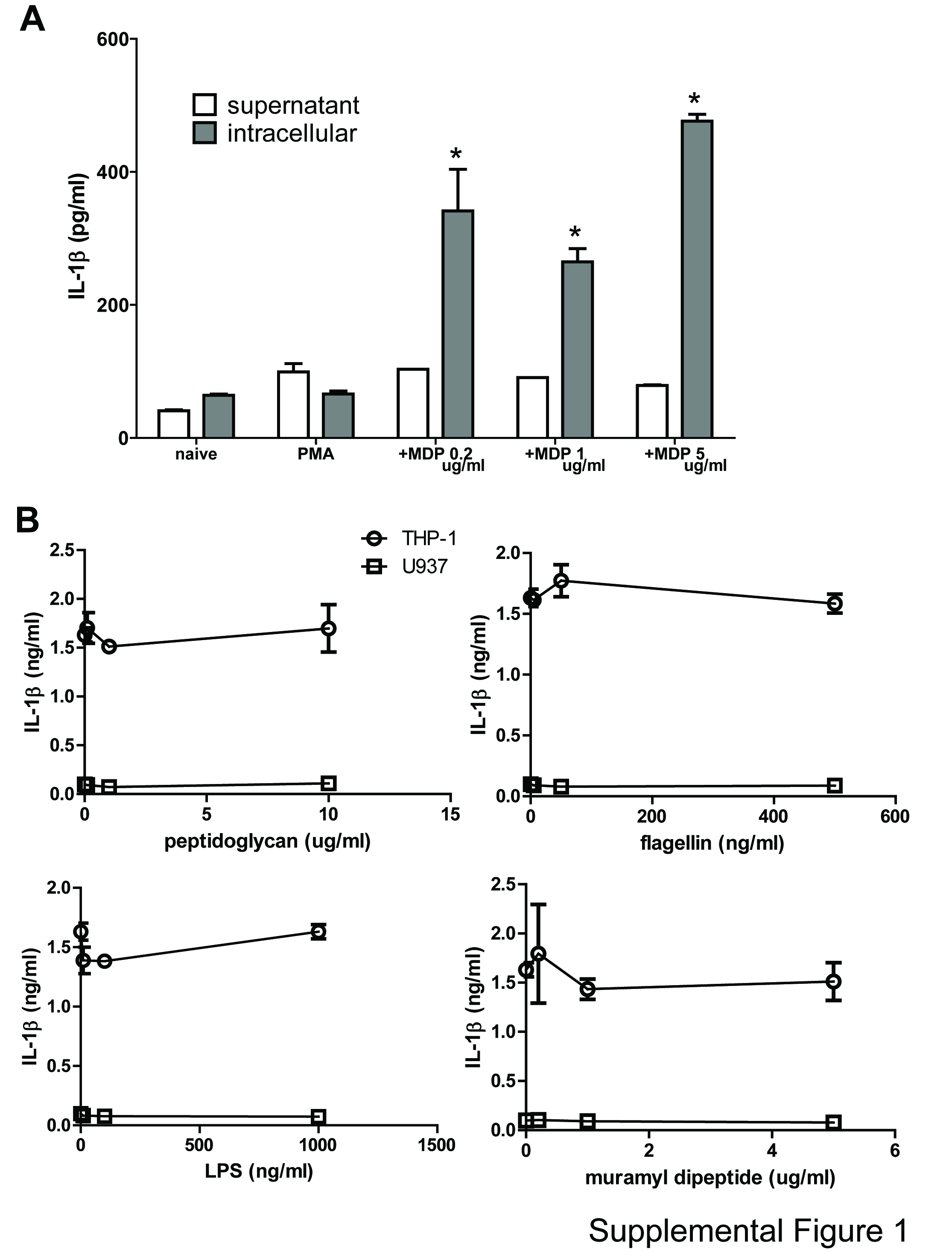

Supplement: Figure S1 — IL-1β Release Compared in U937 and THP-1 Monocytes. A) U937 cells were cultured for 5 days with PMA or PBS control, followed by addition of graded concentrations of muramyl dipeptide (MDP). After 18 hours, supernatants were collected, and cells were washed twice in PBS then lysed. IL-1β was assessed separately in supernatants (open bars) and cell lysates (filled bars) by ELISA. Asterisks denote increase from untreated cells, P≤0.05. B) THP-1 (connected circles) and U937 (connected squares) cultured with PMA for 5 days were pulsed for 18 hours with graded concentrations of TLR ligands peptidoglycan, flagellin, LPS, or MDP. IL-1β was assessed in supernatants by ELISA. (TIF) [file pone.0051078.s001.tif]

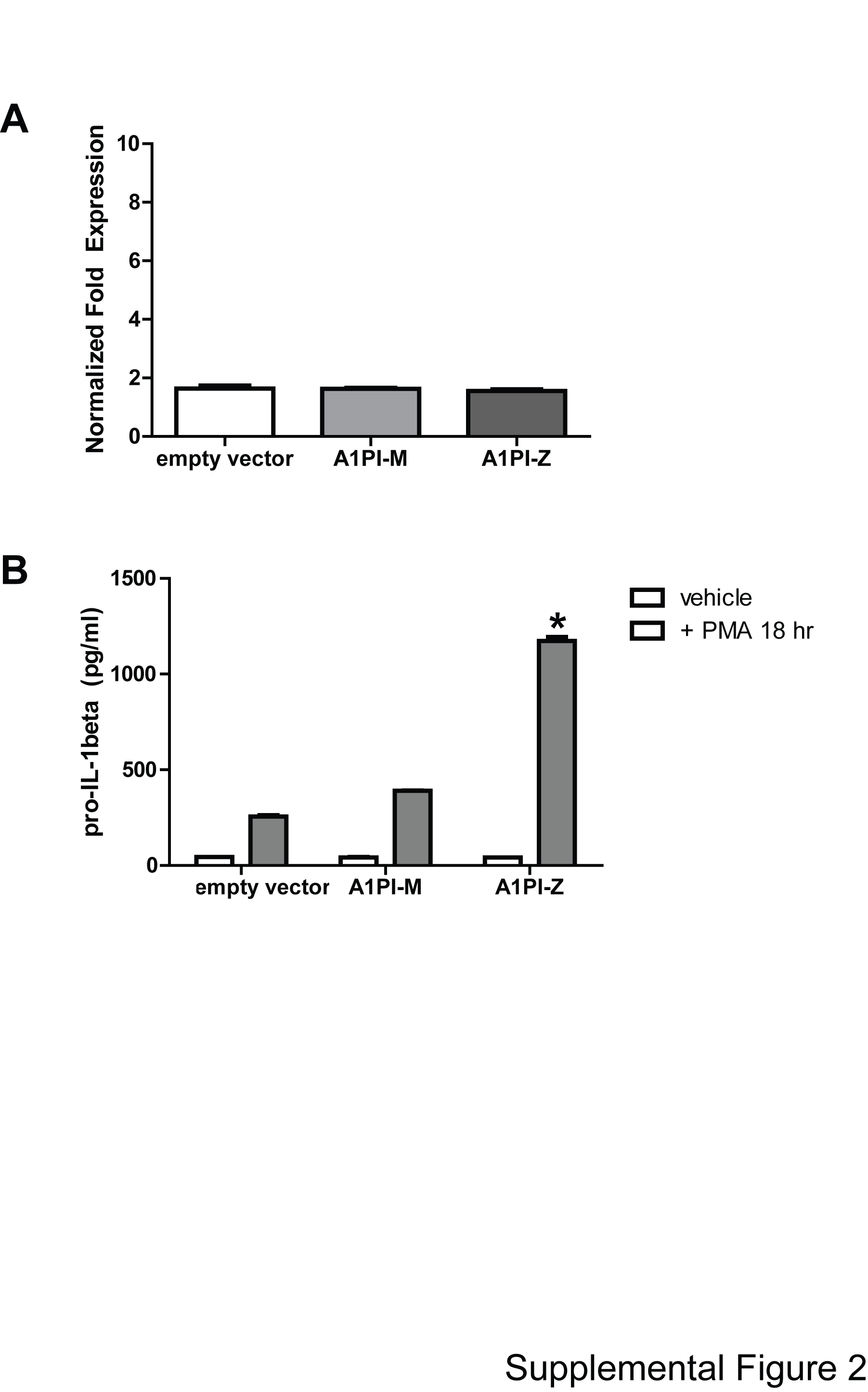

Supplement: Figure S2 — Pro-IL-1β and IL-1β mRNA Detection in Transfected THP-1 Cells. THP-1 cells stably transfected with plasmids encoding wild type A1PI (A1PI-M) or Z isoform A1PI (A1PI-Z), or empty vector control plasmid, were treated for 18 hours with PMA or DMSO vehicle. A) Level of IL-1β mRNA normalized to β-actin was detected by Q-PCR; mean of triplicate wells is shown. B) Level of pro-IL-1β protein in whole cell lysate was detected by ELISA; mean of duplicate wells is shown. Asterisks denote P≤0.05. (TIF) [file pone.0051078.s002.tif]

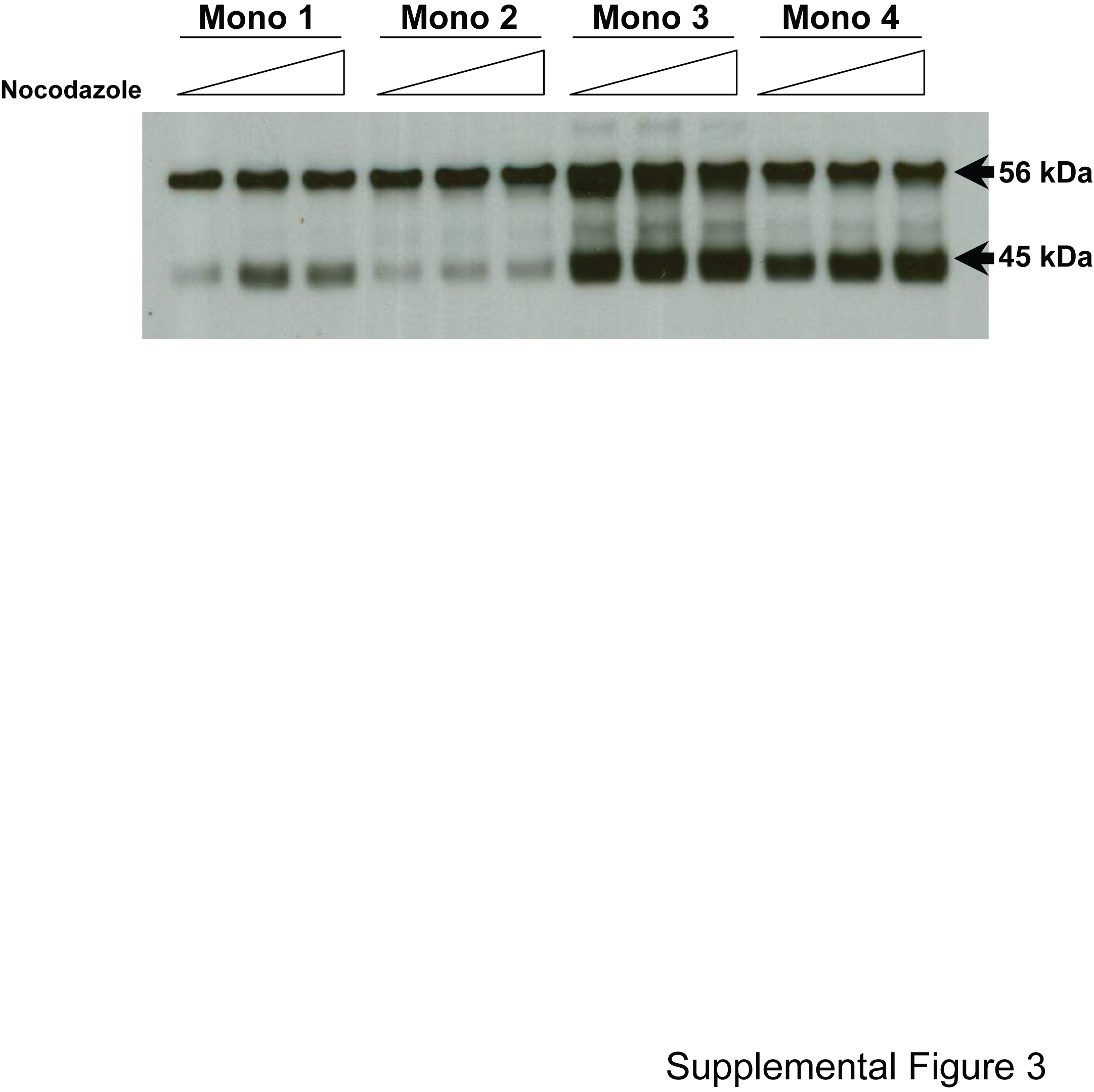

Supplement: Figure S3 — Cytosolic A1PI is Detected after Endocytosis Inhibition in Primary Peripheral Blood Monocytes. Primary elutriated monocytes from healthy adult volunteers were cultured with low or high concentrations of nocodazole plus PMA stimulation for 18 hours. A1PI was detected by immunoblot in cytosolic samples prepared by hypotonic lysis. (TIF) [file pone.0051078.s003.tif]
